# NDRG2 Ameliorates Hepatic Fibrosis by Inhibiting the TGF-β1/Smad Pathway and Altering the MMP2/TIMP2 Ratio in Rats

**DOI:** 10.1371/journal.pone.0027710

**Published:** 2011-11-16

**Authors:** Jiandong Yang, Jin Zheng, Lin Wu, Ming Shi, Hongtao Zhang, Xing Wang, Ning Xia, Desheng Wang, Xinping Liu, Libo Yao, Yan Li, Kefeng Dou

**Affiliations:** 1 Department of Hepatobiliary Surgery, Xijing Hospital, The Fourth Military Medical University, Xi'an, China; 2 Department of Biochemistry and Molecular Biology, State Key Laboratory of Cancer Biology, The Fourth Military Medical University, Xi'an, China; 3 Department of Traditional Chinese and Western Medicine of Oncology, Tangdu Hospital, The Fourth Military Medical University, Xi'an, China; 4 Department of Neurology, Xijing Hospital, The Fourth Military Medical University, Xi'an, China; Chang Gung University, Taiwan

## Abstract

Liver fibrosis is a worldwide clinical issue. It has been well established that activated hepatic stellate cells (HSCs) are responsible for excessive extracellular matrix (ECM) deposition in chronically damaged livers. The identification of key elements that control HSCs activation will help to further our understanding of liver fibrosis and improve the outcome of clinical treatment. This study demonstrates that N-Myc downstream-regulated gene 2 (NDRG2) is a potential regulator of liver fibrosis as NDRG2 mRNA and protein levels were reduced during HSCs activation. In addition, enhanced NDRG2 expression reduced *Smad3* transcription and phosphorylation, which inhibited HSCs activation by blocking the TGF-β1 signal. Moreover, NDRG2 contributed to an increase in the ratio of matrix metalloproteinase 2 (MMP2) to tissue inhibitor of matrix metalloproteinase 2 (TIMP2), which may facilitate the degradation of the ECM. In dimethylnitrosamine (DMN)-induced fibrotic rat livers, adenovirus-mediated NDRG2 overexpression resulted in decreased ECM deposition and improved liver function compared with controls. In conclusion, the present findings indicate that the modulation of NDRG2 is a promising strategy for the treatment of liver fibrosis.

## Introduction

Liver fibrosis is a major medical problem of liver diseases, especially in Asian countries. Chronic hepatitis, metabolic disorders, genetic mutations and cholestatic diseases are common causes of liver fibrosis and even cirrhosis and hepatocellular carcinoma (HCC) [1], [2]. Substantial improvements in the treatment of liver fibrosis have been achieved due to continued studies [3], [4], [5], thereby prompting us to explore the mechanisms involved in the development of liver fibrosis and potential therapies that could inhibit the progression of fibrosis.

HSCs play a central role in liver fibrosis. In normal liver, HSCs are in a quiescent state and their main function is to store retinoids. However, during the development of hepatic fibrosis, chronic liver damage leads to HSCs activation, characterized by a transformation from the quiescent state to a proliferative, contractile and fibrogenic myofibroblast-like phenotype that terminates in excessive hepatic matrix deposition, liver function impairment, cirrhosis and organ failure [6], [7].

The activation of HSCs is finely regulated by multiple pathways and factors, and of these, TGF-β1/Smad signaling is one of the major pathways in charge of HSCs activation, type I collagen expression and deposition. TGF-β1 binds to its receptor, leading to the phosphorylation of the intracellular mediators Smad2 and Smad3, which then form hetero-oligomers with a common mediator, Smad4. The complex then translocates from the cytoplasm to the nucleus to regulate gene transcription. The expression of inhibitory Smad7, which interacts with a group of ubiquitin ligases termed Smurf and degrades the TGF-β receptors through proteasomal and lysosomal pathways, is also induced by TGF-β signaling as part of a negative feedback loop [8], [9]. In addition, TGF-β1 regulates the expression of matrix metalloproteinases (MMPs) and tissue inhibitor of matrix metalloproteinases (TIMPs). MMPs are endogenous peptidases capable of degrading various components of the basement membrane while TIMPs inhibit collagen degradation. In the fibrotic liver, the expression of MMPs and TIMPs are both increased [10] and it is the balance of MMPs and their tissue inhibitors that determines the progression and regression of ECM accumulation and liver fibrosis [11], [12].

Recently, several studies have revealed that NDRG2 is a potent factor in regulating liver embryonic development, tissue remodeling and carcinogenesis. *NDRG2* (GenBank Accession No. AF159092) belongs to the *NDRG* family, which comprises four members, *NDRG1-4*. Our laboratory initially identified human NDRG2, a cytoplasmic protein that is down-regulated by MYC and is involved in cell growth and differentiation, stress and hormonal responses [13], [14], [15]. Accumulated data suggest that NDRG2 is closely involved in liver histogenesis and organogenesis as NDRG2 mRNA and protein levels are generally lower in the early stages and markedly higher during the later stages of histogenesis in mouse and human fetal livers of different gestational ages [16], [17]. Our previous study demonstrated that NDRG2 could regulate liver regeneration by serving as a cell cycle and apoptosis regulator. As a new tumor suppressor gene [18], [19], [20], *NDRG2* also plays a critical role in HCC, in which it is significantly down-regulated compared to adjacent normal tissues. Furthermore, high NDRG2 expression levels correlate positively with tumor differentiation and negatively with clinical parameters relevant to tumor metastasis. *In vitro,* it has been shown that NDRG2 affects the proliferative abilities of HCC cell lines [21] and antagonizes TGF-β1–mediated HCC cell invasion by down-regulating MMP2 [22]. Additionally, when determine the gene expression profile for the whole liver during development of DMN-induced hepatic fibrosis, Takahara et al [23] found that NDRG2 was down-regulated in hepatocytes following fibrogenesis. Taken together, these data implicate the multiple functions of NDRG2 in liver under both normal and pathological conditions. However, to date, the significance of NDRG2 in the development of liver fibrosis has been little studied.

In the current study, we demonstrated that NDRG2 expression exhibited an inverse relationship with HSCs activation. In addition, we have shown that NDRG2 inhibited basal and TGF-β1–mediated HSCs activation via a reduction in Smad3 phosphorylation. Furthermore, adenovirus-mediated NDRG2 overexpression attenuated rat liver fibrosis and improved liver function by enhancing ECM degradation via the alteration of MMP2 and TIMP2 expression. In conclusion, our study is the first to explore the anti-fibrotic effects of NDRG2 in liver.

## Results

### NDRG2 shows an inverse relationship with HSCs activation

**Figure 1:**
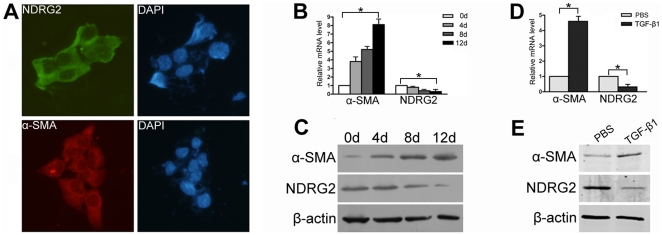
NDRG2 is decreased during LX-2 activation. (A) Green (FITC conjugated) represented NDRG2 expression while red (CY3 conjugated) represented α-SMA expression in LX-2 cells, DAPI (blue)-stained nuclei (400×). Real-time PCR (B) and Western blotting (C) were used to detect the expression of α-SMA and NDRG2 in LX-2 cells grown to confluence and then replated on 100 mm dishes for 12 days. Real-time PCR (D) and Western blotting (E) were used to detect the expression of α-SMA and NDRG2 in LX-2 cells following serum starvation and supplementing with 0.2% BSA for 48 hours prior to PBS or TGF-β1 treatment (2.5 ng/ml for 24 hours). β-actin was used as a loading control. All experiments were performed independently in triplicate. *P<0.05 between compared groups.

The spontaneously immortalized human HSCs cell line, LX-2, exhibits features typical of stellate cells, while the immortalized rat HSCs cell line, HSC-T6, exhibits features typical of myofibroblasts [24]. In this study, NDRG2 was expressed in LX-2 cells and was mainly localized to the cytoplasm, together with alpha smooth muscle actin (α-SMA), a marker of HSCs activation (Figure 1A). Previous studies have demonstrated that HSCs undergo further activation during growth and expansion on a plastic surface [25], and using this model we were able to assess the relationship between NDRG2 expression and LX-2 cell activation. After LX-2 cells grew to confluence, they were trypsinized and replated in 100 mm dishes for further activation. Over the next 12 days, increased expression of α-SMA was detected, indicating LX-2 activation while, in contrast, NDRG2 mRNA and protein expression decreased (Figure 1B and C). Similar results were observed in HSC-T6 cells (Figure S1).

TGF-β1 is a classic stimulator for HSCs activation and collagen production [26], [27]. To confirm the relationship between NDRG2 and HSCs activation further, LX-2 cells were treated with TGF-β1 for 24 h after serum starvation. As expected, TGF-β1 increased LX-2 activation, as indicated by enhanced α-SMA levels compared to controls, while NDRG2 expression was inhibited (Figure 1D and E).

### NDRG2 inhibits HSCs activation

**Figure 2:**
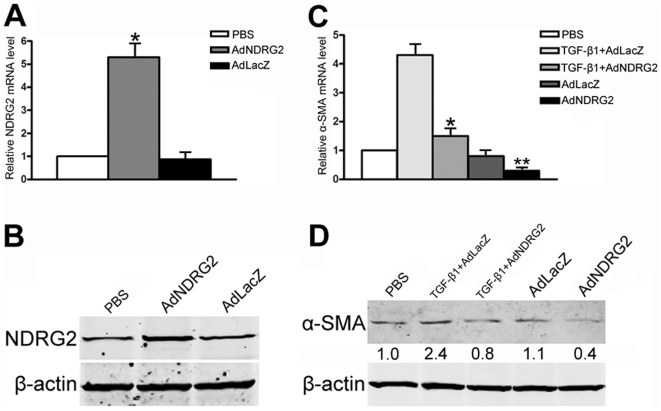
NDRG2 inhibits HSCs activation. NDRG2 mRNA (A) and protein (B) level in LX-2 cells assessed 48 hours after PBS or adenovirus infection. α-SMA mRNA (C) and protein (D) level in LX-2 cells assessed after PBS, TGF-β1 and/or adenovirus infection. β-actin was used as a loading control. Relative quantifications of α-SMA band intensities, normalized to β-actin levels, were shown below blots. All experiments were performed independently in triplicate. In (A), *P<0.05 compared with the PBS or AdLacZ group. In (C), *P<0.05 compared with the TGF-β1+AdLacZ group; **P<0.05 compared with the AdLacZ or PBS group.

Since NDRG2 was down-regulated during HSCs activation, adenovirus vectors were used to elevate NDRG2 expression transiently in LX-2 cells to determine the effect of NDRG2 on HSCs activation. The results showed that adenoviral vectors expressing *NDRG2* (AdNDRG2) enhanced the level of NDRG2 compared to the negative control β-galactosidase (AdLacZ) group (Figure 2A and B). Next, the activation level of LX-2 cells was examined by treating the cells with adenovirus and/or TGF-β1. After treatment, both the basal and TGF-β1–induced α-SMA protein levels were decreased by NDRG2 overexpression (Figure 2C and D). These results indicated that NDRG2 could inhibit HSCs activation, even when mediated by TGF-β1.

### NDRG2 inhibits the TGF-β1/Smad signaling pathway and alters the relative levels of MMP2 to TIMP2

**Figure 3:**
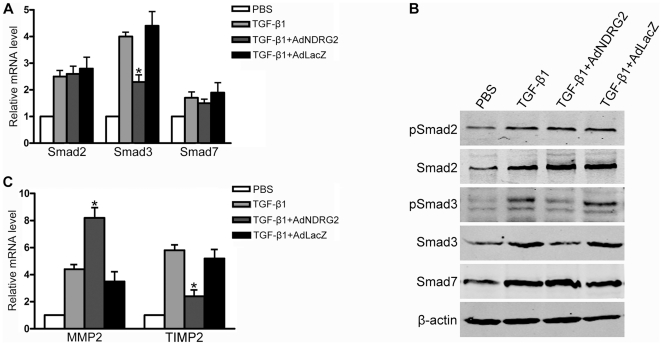
NDRG2 affects TGF-β1/Smad signaling pathway and the ratio of MMP2 to TIMP2 in LX-2 cells. (A) Real-time PCR was used to detect the expression of Smad2, Smad3 and Smad7 in LX-2 cells with TGF-β1 and/or adenovirus treatment. (B) Western blotting was used to detect the expression of Smad2, phospho-Smad2, Smad3, phospho-Smad3 and Smad7 in LX-2 cells with TGF-β1 and/or adenovirus treatment. (C) Real-time PCR was used to detect the expression of MMP2 and TIMP2 in LX-2 cells following TGF-β1 and/or adenovirus treatment. β-actin was used as a loading control. All experiments were performed independently in triplicate. *P<0.05 compared with the TGF-β1 or TGF-β1+AdLacZ group.

TGF-β1 signaling is essential for HSCs activation and liver fibrosis. To gain a better understanding of the molecular mechanisms involved in the NDRG2-mediated inhibition of HSCs activation, the mRNA levels of the intracellular mediators of TGF-β1 signal transduction *Smad2*, *Smad3* and *Smad7* were analyzed. The results demonstrated that the transcription of all three Smads increased in TGF-β1–treated LX-2 cells; however, AdNDRG2 only attenuated the increase in *Smad3* induced by TGF-β1, while *Smad2* and *Smad7* showed no differences among compared groups (Figure 3A). Furthermore, the protein levels of Smad2/3/7 as well as the phosphorylation of Smad 2/3 were upregulated in the presence of TGF-β1 stimulation, while AdNDRG2 treatment only inhibited the protein level and the phosphorylation status of Smad3 (Figure 3B).

MMPs and TIMPs contribute to both the progression and regression of liver fibrosis. In this study, the expression of both MMP2 and TIMP2 increased during LX-2 activation induced by TGF-β1; however, after AdNDRG2 infection, MMP2 was enhanced while TIMP2 levels decreased (Figure 3C) compared with AdLacZ controls (MMP9 and TIMP1 were undetectable). Consequently, the ratio of MMP2 to TIMP2 was increased (Table 1), which is helpful for the regression of ECM accumulation.

**Table 1:** NDRG2 affects the ratio of MMP2 to TIMP2 in LX-2 cells.

Group	MMP2/TIMP2
PBS	1
TGF-β1	0.764±0.0003
TGF-β1+AdNDRG2	3.418±0.3124*
TGF-β1+AdLacZ	0.669±0.0639

*P<0.05 compared with the TGF-β1 or TGF-β1+AdLacZ group.

### NDRG2 attenuates DMN-induced liver fibrosis

**Figure 4:**
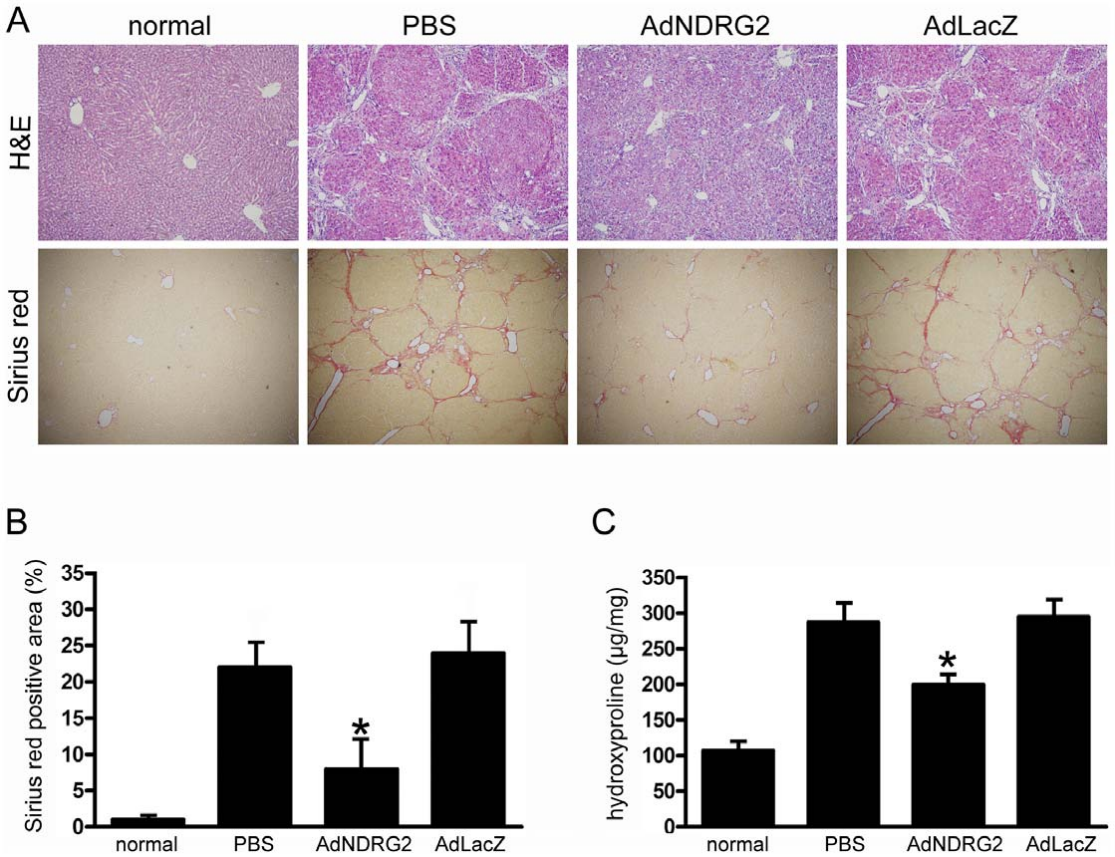
NDRG2 attenuates DMN-induced liver fibrosis. PBS or adenovirus was injected four weeks after DMN treatment and rats were sacrificed two weeks after injection. (A) Liver fibrosis was assessed by H&E staining (100×) and Sirius Red staining (40×). (B) Semiquantitative analysis of the Sirius Red staining result. (C) Assay of hydroxyproline content. The data are expressed as hydroxyproline (µg)/wet liver weight (mg). Three samples were determined from each group. “normal” means rats without treatment. *P<0.05 compared with PBS or AdLacZ group.

**Figure 5:**
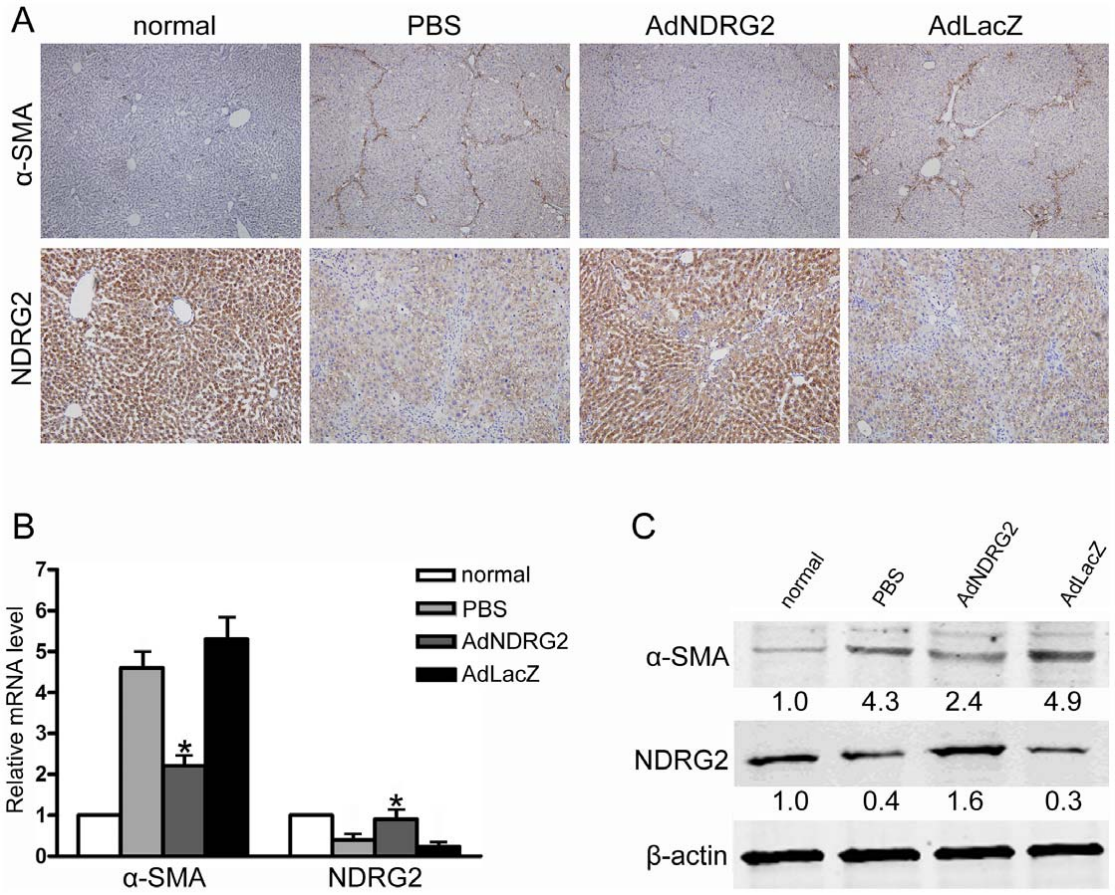
NDRG2 inhibits HSCs activation in DMN-induced liver fibrosis. (A) Immunohistochemistry was used to determine the expression of NDRG2 (200×) and α-SMA (100×). Real-time PCR (B) and Western blotting (C) were used to detect the expression of α-SMA and NDRG2 in liver tissues. β-actin was used as a loading control. Relative quantifications of band intensities, normalized to β-actin levels, were shown below blots. “normal” means rats without treatment. *P<0.05 compared with the PBS or AdLacZ group.

To explore the anti-fibrosis role of NDRG2 *in vivo*, a rat liver fibrosis model was established by DMN injection. Rats receiving DMN treatment developed severe fibrosis as shown in a representative hematoxylin and eosin (H&E) and Sirius Red stained image (Figure 4A). In addition, the expression of NDRG2 in liver cells decreased while α-SMA positive cells increased dramatically (Figure 5A), indicating that HSCs had been activated following DMN treatment.

To determine the infection efficiency of the adenovirus, rats were injected first with phosphate buffered saline (PBS) or a single dose of adenoviral vector (4×10^9^ plaque forming units [PFU]) expressing enhanced green fluorescent protein (AdEGFP) via the tail vein. Forty-eight hours after injection, over 80% of liver cells were EGFP-positive compared with the PBS group (Figure S2A), showing that the adenovirus had a high infection efficiency. In addition, we found that after administration of the same dose of AdNDRG2, NDRG2 levels were significantly enhanced in rat liver from day three to day 14 (Figure S2B) while AdLacZ treatment had no effect on NDRG2 expression (Figure S2C). Thus, a single dose of adenovirus was enough to enhance NDRG2 expression, which lasted long enough to produce a therapeutic effect on liver fibrosis.

After treatment, we observed that, compared with PBS or AdLacZ injection, AdNDRG2 administration not only elevated NDRG2 expression in liver cells but also inhibited the expression of α-SMA (Figure 5), which demonstrated further that NDRG2 could suppress HSCs activation. In addition, the degree of fibrosis was significantly attenuated as revealed by H&E and Sirius Red staining (Figure 4A and B). Meanwhile, after AdNDRG2 injection, the hydroxyproline content, which is an indicator for ECM deposition, was decreased in the AdNDRG2-treated fibrotic livers (199.69±8.23 µg/mg) compared with the AdLacZ group (295.06±17.14 µg/mg) or the PBS group (287.46±20.09 µg/mg) (Figure 4C).

**Figure 6:**
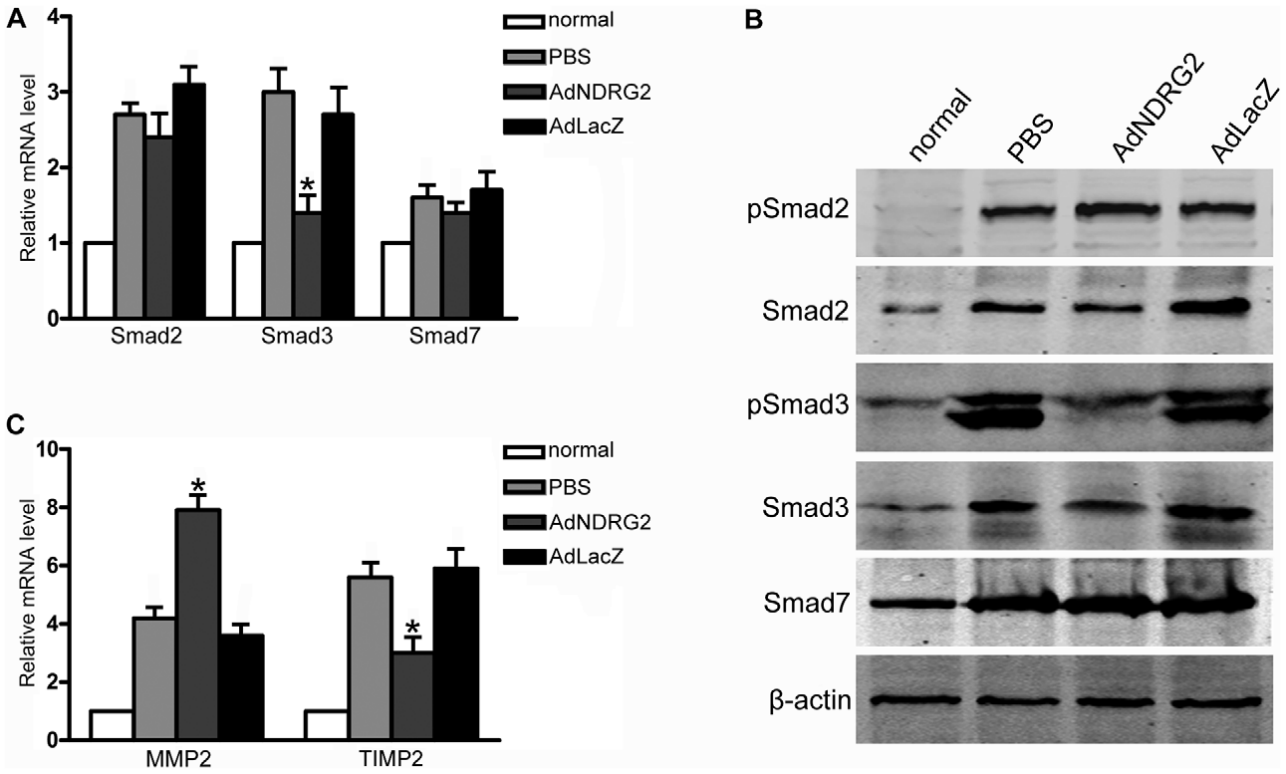
NDRG2 affects the TGF-β1/Smad signaling pathway and the ratio of MMP2 to TIMP2 in rats. (A) Real-time PCR was used to detect the expression of Smad2, Smad3 and Smad7 in rats with PBS or adenovirus treatment. (B) Western blotting was used to detect the expression of Smad2, phospho-Smad2, Smad3, phospho-Smad3 and Smad7 in rats with PBS or adenovirus treatment. (C) Real-time PCR was used to detect the expression of MMP2 and TIMP2 in rats following PBS or adenovirus treatment. β-actin was used as a loading control. “normal” means rats without treatment. *P<0.05 compared with the PBS or AdLacZ group.

In line with the *in vitro* results, the data obtained from fibrotic rats indicated that, following AdNDRG2 treatment, the expression and phosphorylation of Smad3 decreased while the ratio of MMP2 to TIMP2 increased (Figure 6) (Table 2), confirming that the regulatory role of NDRG2 in TGF-β1/Smad signaling and in the regulation of MMP2 and TIMP2 expression contributes towards its therapeutic effects in the fibrotic liver.

**Table 2:** NDRG2 affects the ratio of MMP2 to TIMP2 in rats.

Group	MMP2/TIMP2
normal	1
PBS	0.749±0.0076
AdNDRG2	2.640±0.2891*
AdLacZ	0.615±0.0125

*P<0.05 compared with the PBS or AdLacZ group. “normal” means rats without treatment.

### NDRG2 increases hepatocyte proliferation and improves liver function

**Figure 7:**
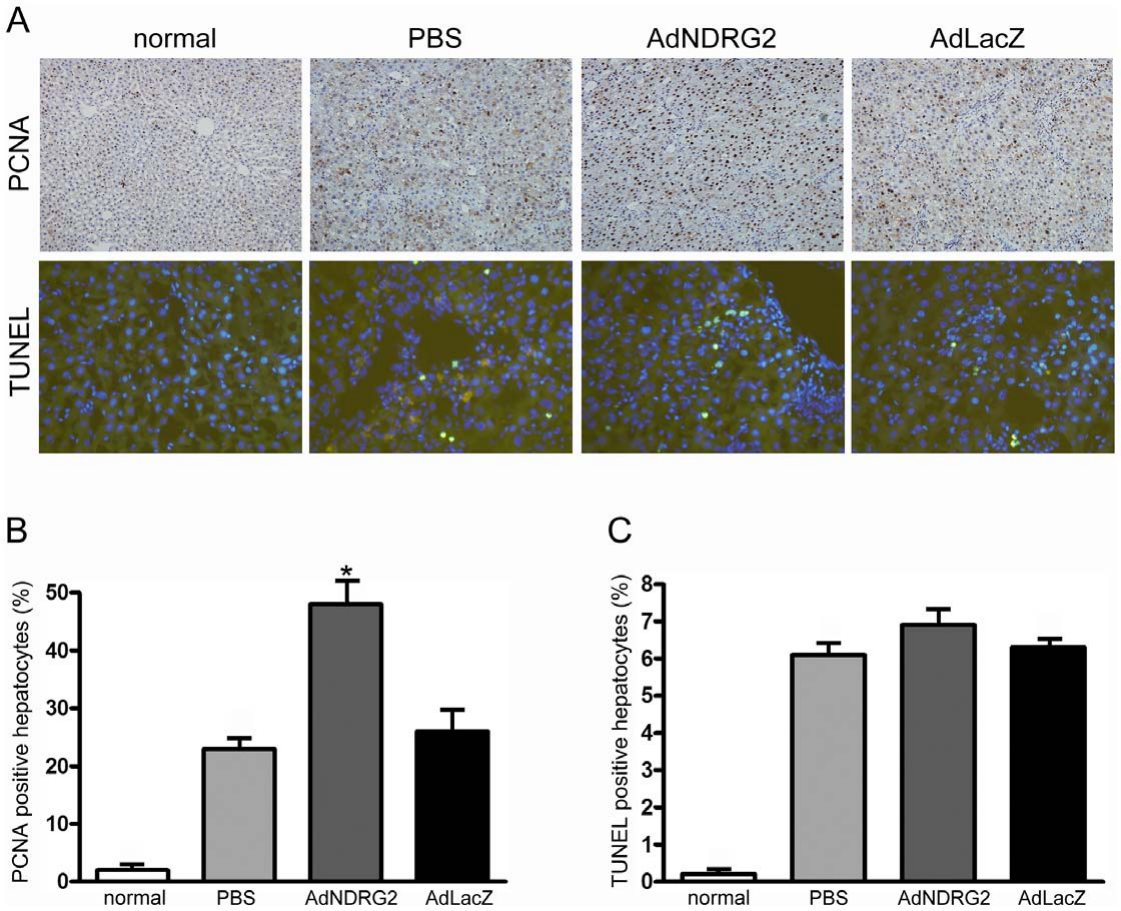
NDRG2 promotes hepatocyte proliferation without inducing apoptosis. (A) Immunohistochemistry and TUNEL staining were used to determine the expression of PCNA (200×) and cell apoptosis (400×) in normal and DMN-induced fibrotic rat livers following PBS or adenovirus treatment. Semiquantitative analysis of PCNA staining (B) and TUNEL staining (C). “normal” means rats without treatment. *P<0.05 compared with the PBS or AdLacZ group.

Our previous data demonstrated that AdNDRG2 treatment induced hepatocyte cell cycle arrest and apoptosis *in vitro* [28]. However, in the present study, it is important to note that hepatocytes responded differently to AdNDRG2 in DMN-induced liver fibrosis. Proliferating cell nuclear antigen (PCNA) staining and BrdU staining increased in AdNDRG2 group compared with PBS or AdLacZ group while TUNEL analysis revealed that AdNDRG2 had no effect on inducing hepatocyte apoptosis (Figure 7) (Figure S3), which indicating that AdNDRG2 not only inhibited HSCs activation and ECM accumulation, but also facilitated the regression of liver fibrosis by enhancing the proliferation of hepatocytes without inducing apoptosis. Moreover, AdNDRG2 injection significantly improved hepatic serum biochemical parameters in the DMN-induced rat fibrotic model (Table 3).

**Table 3:** NDRG2 improves hepatic serum biochemical parameters.

Group	ALB(g/l)	ALT(U/l)	AST(U/l)
normal	36.5±3.2	43.1±7.2	92.4±14.9
PBS	15.4±1.6	208.2±9.8	251.4±32.7
AdNDRG2	26.6±4.8*	156.6±25.7*	173.8±10.0 *
AdLacZ	13.1±0.7	218.7±12.4	279.2±35.5

*P<0.05 compared with the PBS or AdLacZ group. “normal” means rats without treatment.

## Discussion

HSCs can undergo further activation either by cultured on uncoated plastic or treated with TGF-β1. These two models are well-established techniques for studying hepatic fibrogenesis at the cellular level. The present data demonstrated a decrease in NDRG2 expression using both of these methods for HSCs activation. Previous studies have revealed that the level of NDRG2 expression is linked positively to cell differentiation and negatively to cell proliferation [16], [17], [29], [30], [31], and it is well known that during chronic liver injury, HSCs activation involves the process of transdifferentiating from quiescent into proliferative and ECM-producing myofibroblasts [32]. Thus, the decrease in NDRG2 levels during the HSCs activation process is another evidence for the regulatory role of NDRG2 on cell differentiation and proliferation. Taking this into account, whether or not NDRG2 regulates the proliferative ability and transdifferentiation process of HSCs merits further investigation.

TGF-β1 is a profibrogenic cytokine in the liver. The current study has shown that NDRG2 inhibited HSCs activation in the absence and presence of TGF-β1, and in addition, increased the expression of MMP2 in HSCs. These results differ partly from research into HCC, which revealed that overexpression of NDRG2 antagonized TGF-β1–mediated HCC invasion by down-regulating MMP2 expression [22]. However, the previous study was based on the HCC cell line PLC/PRF/5. Most cases of HCC develop on a liver fibrosis background [33] and a fibrous capsule surrounds a typical HCC at an early stage [34]. HCC cells have to degrade the ECM and basement membrane during the process of metastasis [35]. Therefore, overexpression of NDRG2 leads to reduced MMP2 and ECM degradation, which subsequently, confined HCC to its fibrous septa. Yet, in our study, AdNDRG2 treatment produced an increase in MMP2 as well as a decrease of TIMP2. MMP2 activity is regulated at the cell surface and involves the formation of a tri-molecular complex consisting of pro-MMP2, MT1-MMP and TIMP-2. All three components of this complex are expressed by activated HSCs [36]. An increase in MMPs and TIMPs is common in fibrotic diseases, while an alteration in the MMP/TIMP expression ratio is known to be critical in determining matrix remodeling [10]. In this study, the data from LX-2 cells demonstrated that over-expressing NDRG2 inhibited the expression of TIMP2 and increased MMP2. Consequently, NDRG2 administration reduced ECM deposition within the liver parenchyma and alleviated liver fibrosis.

Considering the significance of the TGF-β1/Smad signaling pathway in regulating fibrogenesis, researchers are trying to block the TGF-β1/Smad signal in order to suppress liver fibrosis. Importantly, Smad2 and Smad3 exert their functions depending on the status of HSCs. TGF-β1 induces the phosphorylation and nuclear translocation of Smad2 in quiescent HSCs and of Smad3 in transdifferentiated HSCs [37]. In addition, data obtained from infecting cultured rat HSCs with adenoviruses expressing *Smad2* or *Smad3* revealed that Smad3 plays a more important role than Smad2 in the morphological and functional maturation of myofibroblasts [38]. In the present study, LX-2 cells could be regarded as partially activated based on the fact that they expressed α-SMA under all culture conditions [25]. By infecting LX-2 cells with adenovirus expressing NDRG2, we found that an increase in NDRG2 in LX-2 cells caused a decrease in Smad3 and its phosphorylation in the presence of TGF-β1. In contrast, the status of Smad2 and Smad7 were not affected. Data obtained from fibrotic rat livers were also consistent with the *in vitro* study. Therefore, the decreased activation and inhibited transdifferentiation of HSCs depend on the suppression of Smad3 after AdNDRG2 treatment.

A recent study from our laboratory revealed that the overexpression of NDRG2 in a hepatocyte cell line resulted in G_1_/S arrest and apoptosis induction. During the early phase of liver regeneration in a rat partial hepatectomy (PH) model, NDRG2 was down-regulated, which may have facilitated hepatocyte proliferation [28]. Based on these data, we wondered whether AdNDRG2 treatment could inhibit hepatocyte proliferation and cause apoptosis in fibrotic rat liver, which were harmful for liver fibrosis treatment. However, in our current study, enhanced PCNA and BrdU staining in the fibrotic liver implied that AdNDRG2 injection increased the proliferation of hepatocytes. In addition, TUNEL analysis revealed that there were no significant differences between the numbers of apoptotic cells in the AdNDRG2 group and the PBS and AdLacZ-treated groups. The discrepancies between these two studies may be attributed to the complexity of the NDRG2 regulation network in different liver tissue remodeling microenvironments (PH-induced liver regeneration compared to DMN-induced liver fibrosis). In addition, both TGF-β1 and ECM deposition have an anti-proliferative and pro-apoptotic effect on hepatocytes [39]. Thus, in the current study, it is possible that AdNDRG2 treatment exerted its therapeutic function by antagonizing TGF-β1 and subsequently increasing hepatocyte proliferation without inducing cell apoptosis, processes essential for treating liver fibrosis and improving liver function.

Collectively, the data reported in this study have demonstrated, for the first time, that NDRG2 is involved in the regulation of liver fibrosis. NDRG2 expression correlated inversely with HSCs activation and, in addition, inhibited TGF-β1–induced HSCs activation by suppressing Smad3 expression and phosphorylation. Furthermore, the *in vivo* study indicated that adenovirus-mediated NDRG2 treatment could effectively attenuate liver fibrosis and improve liver function. Thus, it is conceivable that modulating NDRG2 expression may provide a novel therapy for intervention in liver fibrosis.

## Materials and Methods

### Cell lines and cell culture

LX-2 and HSC-T6 cells [24] were kindly provided by Dr Scott Friedman. Cells were maintained in Dulbecco's modified Eagle's medium (DMEM, Invitrogen Life Technologies, Carlsbad, CA, USA) supplemented with 10% fetal bovine serum (FBS, Invitrogen) in a humidified atmosphere of 5% CO_2_ at 37°C.

### Immunofluorescence analysis

Cells were seeded in 24-well plates and treated with 4% paraformaldehyde for 15 min at 4°C. After blocking with 1% bovine serum albumin (BSA) in PBS containing 0.1% Triton X-100 for 1 hour at room temperature, the cells were incubated with primary NDRG2 (Abnova, Taiwan, China) or α-SMA (Abcam, Cambridge, MA, USA) antibodies overnight at 4°C, followed by three washes with PBS and incubation for 2 hours with FITC- or CY3-conjugated secondary antibodies (Boster, Wuhan, China). Cell nuclei were stained with 4,6-diamidino-2-phenylindole (DAPI; Molecular Probes, OR).

### TGF-β1 treatment

Cells were plated in 100 mm dishes. Once they reached 70% confluence, the cells were serum starved and supplemented with 0.2% BSA for 48 hours. Next, TGF-β1 (2.5 ng/ml, PeproTech, Rocky Hill, NJ, USA) was added for 24 hours.

### Adenovirus infection

Adenoviral vectors expressing human or rat *NDRG2* (AdNDRG2), the negative control, β-galactosidase (AdLacZ), and enhanced green fluorescent protein (AdEGFP) were purchased from Benyuan Zhengyang Company (Beijing, China). Cells were seeded in 100 mm dishes and treated with AdNDRG2, AdLacZ or PBS in serum-free DMEM for 2 hours. The medium was replaced with fresh DMEM supplemented with 10% FBS and incubated for 48 hours. The multiplicity of infection (MOI) was 40, which was determined according to preliminary experimental results.

### Animals

Seven-week-old male Sprague-Dawley rats weighing between 200 and 225 g were housed individually in cages with a 12 hour light-dark cycle and given free access to water and standard rat chow throughout the study. All experimental procedures were conducted in accordance with the ‘Detailed Rules for the Administration of Animal Experiments for Medical Research Purposes’ issued by the Ministry of Health of China and had received ethical approval (Permit number: SCXK 2007-007) by the Animal Experiment Administration Committee of the Fourth Military Medical University (Xi'an, P.R., China). All efforts were made to minimize animals' suffering and to reduce the number of animals used.

### Fibrosis model and tissue preparation

Rats were injected intraperitoneally with 1% DMN (10 µg/kg) for three consecutive days per week for up to five weeks. After 12 DMN injections, rats were infused with PBS or a single dose of 4×10^9^ PFU of AdLacZ or AdNDRG2 via the tail vein (ten rats in each group). Two weeks after gene delivery, animals were sacrificed and liver samples were collected. For 5′-Bromo-2′-deoxyuridine (BrdU) staining, one hour before sacrifice, 100 mg BrdU (Sigma, St. Louis, MO) per kg body weight was injected intraperitoneally. RNA and protein were extracted directly with standard techniques described below as soon as the liver samples were excised. For immunohistochemistry analysis, samples were fixed in 4% phosphate-buffered formalin for two days and then embedded in paraffin before 3 µm sections were cut and collected on glass slides. For apoptosis analysis, samples were fixed with neutral buffered 4% paraformaldehyde overnight at 4°C, and liver sections of 10 µm thickness were prepared using a cryostat microtome (Leica, Solms, Germany) and fixed to poly-L-lysine–coated glass slides. Sections were stored at -20°C until required.

### Immunohistochemistry analysis

Sections were deparaffinized in xylene and dehydrated through a graduated alcohol series. After incubating with 0.3% H_2_O_2_ in methyl alcohol for 15 min, sections were treated with citrate buffer (pH 6.0) for antigen retrieval and incubated with 10% normal goat serum in PBS for 1 hour at room temperature to block nonspecific binding. Then, the sections were incubated with the following primary antibodies in PBS at 4°C overnight: mouse anti-NDRG2 (1∶100; Abnova), mouse anti-PCNA (1∶2000; Cell Signaling Technology, Danvers, MA, USA), rabbit anti-α-SMA (1∶100; Abcam), and mouse anti-BrdU (1∶100; Santa Cruz Biotechnology, CA, USA). After equilibrating to room temperature, sections were first incubated with biotinylated goat anti-mouse/rabbit IgG (1∶400, Sizhengbo, Beijing, China) for 2 hours and then treated with a streptavidin-horseradish peroxidase complex (Sizhengbo) for 1 h at room temperature. Staining was examined by incubation with 3,3′-diaminobenzidine (DAB) and the sections were counterstained with hematoxylin and viewed under a light microscope.

### Sirius Red staining

Sections were deparaffinized and the slides were incubated for 10 min in celestine blue solution and washed with distilled water three times. Next, the slides were placed in a solution of saturated picric acid containing 0.05% Sirius Red for 30 min. After washing with 100% ethanol, slides were mounted in mounting media and covered with a coverslip. The results of Sirius Red staining were quantified with Image-Pro Plus software 6.0 (Media Cybernetics, L.P., Silver Spring, MD).

### Apoptotic cell staining

For apoptosis detection in frozen liver tissue sections, terminal deoxynucleotidyl transferase-mediated deoxyuridine triphosphate nick-end labeling (TUNEL) staining was performed as described in the manufacturer's instructions (Roche, Indianapolis, IN, USA). Staining was visualized by fluorescence microscopy (Olympus, Center Valley, PA).

### Hepatic function and hydroxyproline content

An automated analyzer at the Department of Pharmacology in the Fourth Military Medical University was used to analyze serum biochemical parameters, and 100 mg of wet liver samples were subjected to acid hydrolysis to measure the hydroxyproline content according to the Hydroxyproline Testing Kit (Jiancheng, Nanjing, China) protocol.

### Real-time PCR

Total RNA was isolated from each sample using TRIzol reagent (Invitrogen) and then quantified. The cDNA was synthesized from 5 µg of each RNA sample with a Taqman reverse transcriptase reagent kit (Applied Biosystems, UK) primed with oligo(dT) and was then used as a template for real-time quantitative PCR analysis. The sequences of the primers are listed in Table 4. The mRNAs were detected with SYBR Green PCR Master Mix and an ABI PRISM 7500 Sequence Detection System (Applied Biosystems) using the comparative threshold cycle method for relative quantification. The PCR reaction consisted of 12.5 µl SYBR Green PCR Master Mix, 10 pmol of the forward and reverse primers, and 5 µl of template cDNA in a total volume of 25 µl. The thermal cycling conditions comprised an initial denaturation step at 95°C for 10 s, followed by 45 cycles at 95°C for 5 s and 60°C for 34 s.

**Table 4:** Primers for Real-time PCR.

*Genes*	*Forward primer (5′- 3′)*	*Reverse primer (5′- 3′)*
Human NDRG2	GAGATATGCTCTTAACCACCCG	GCTGCCCAATCCATCCAA
α-SMA	GACAATGGCTCTGGGCTCTGTAA	CTGTGCTTCGTCACCCACGTA
Smad2	TTAACCGAAATGCCACGGTAGAA	GCTCTGCACAAAGATTGCACTATCA
Smad3	AGGCGTGCGGCTCTACTACATC	CAGCGAACTCCTGGTTGTTGAA
Smad7	TGCTGTGCAAAGTGTTCAGGTG	CCATCGGGTATCTGGAGTAAGGA
MMP2	ATGACATCAAGGGCATTCAGGAG	TCTGAGCGATGCCATCAAATACA
TIMP2	GACGGCAAGATGCACATCAC	GAGATGTAGCACGGGATCATGG
β-actin	AGCGAGCATCCCCCAAAGTT	GGGCACGAAGGCTCATCATT
Rat NDRG2	ATGGCAGAGCTTCAGGAGG	CGGGTGGTTCAGAGCGTAT
α-SMA	CCGAGATCTCACCGACTACC	TCCAGAGCGACATAGCACAG
Smad2	TTACAGATCCATCGAACTCGGAGA	CACTTAGGCACTCGGCAAACAC
Smad3	CCAGATGAACCACAGCATGGA	CTACTGTCATGGACGGCTGTGAA
Smad7	CCTTACTCCAGATACCCGATGG	CTTGTTGTCCGAATTGAGCTGT
MMP2	TCCCGAGATCTGCAAGCAAG	AGAATGTGGCCACCAGCAAG
TIMP2	GACACGCTTAGCATCACCCAGA	CTGTGACCCAGTCCATCCAGAG
β-actin	ACCGTGAAAAGATGACCCAGAT	AACCCTCATAGATGGGCACAGT

### Western blot analysis

Cells were harvested from 100 mm culture dishes. Both cells and liver tissues were lysed in 200 µl RIPA buffer (0.05 M Tris-HCl [pH 7.4], 0.15 M NaCl, 0.25% deoxycholic acid, 1% Nonidet P-40, 1 mM EDTA, 1 mM phenylmethylsulfonyl fluoride, 10 µg/ml aprotinin and 10 µg/ml leupeptin). Protein concentrations were measured using the bicinchoninic acid (BCA) protein assay (Pierce, Rockford, IL, USA). Proteins were resolved by SDS-PAGE and transferred to Hybond-ECL nitrocellulose membranes (Amersham Biosciences, Piscataway, NJ, USA). The blots were probed with the following primary antibodies: NDRG2 (Abnova), β-actin, Smad7 (Santa Cruz Biotechnology), α-SMA (Abcam), Smad2/phospho-Smad2 (pSmad2), Smad3/phospho-Smad3 (pSmad3) (Cell Signaling Technology), followed by incubation with species-matched secondary antibodies. The bands were detected using enhanced chemiluminescence (Pierce) or the Odyssey Imaging System (Li-Cor Biosciences). Band intensities were quantified with Kodak Digital Science 1D 3.0 (Eastman Kodak, New Haven, CT).

### Statistical analysis

Statistical analyses were performed with SPSS software (version 16.0; SPSS, Chicago, IL, USA) using the t-test and analysis of variance of independent groups. Statistical significance was based on a value of P<0.05.

## Supporting Information

Figure S1 **NDRG2 is decreased during HSC-T6 activation.** (A) NDRG2 was expressed in LX-2 and HSC-T6 cells. Western blotting (B) and real-time PCR (C) were used to detect the expression of α-SMA and NDRG2 in HSC-T6 cells grown to confluence and then replated on 100 mm dishes for 12 days. β-actin was used as a loading control. All experiments were performed independently in triplicate. *P<0.05 between compared groups.
(TIF)

Figure S2 **Adenovirus has a high infection efficiency and significantly enhanced NDRG2 expression in rat liver.** (A) Rats were injected with PBS or a single dose (4×10^9^ PFU) of AdEGFP via the tail vein. Then, 48 hours after injection, rats were sacrificed and liver samples were fixed with neutral buffered 4% paraformaldehyde overnight at 4°C in the dark. Liver sections (10 µm thick) were prepared on a cryostat microtome and fixed to glass slides. Sections were examined directly under a fluorescent microscope (100×). (B) Rats were treated with a single dose (4×10^9^ PFU) of AdNDRG2 or AdLacZ via the tail vein. Over the next 14 days, rats were sacrificed at the time points indicated. Liver samples were collected and proteins were extracted directly. NDRG2 expression was examined by Western blotting. β-actin was used as a loading control.
(TIF)

Figure S3 **NDRG2 promotes hepatocyte proliferation.** (A) Immunohistochemistry staining was used to determine the expression of BrdU (200×) in DMN-induced fibrotic rat livers following PBS or adenovirus treatment. (B) Semiquantitative analysis of BrdU staining. *P<0.05 compared with the PBS or AdLacZ group.
(TIF)
