# Supplementary figures and images for: NDRG2 Ameliorates Hepatic Fibrosis by Inhibiting the TGF-β1/Smad Pathway and Altering the MMP2/TIMP2 Ratio in Rats
Source: PLoS One. 2011 Nov 16;6(11):e27710. doi: 10.1371/journal.pone.0027710 (PMC3218018; doi:10.1371/journal.pone.0027710)

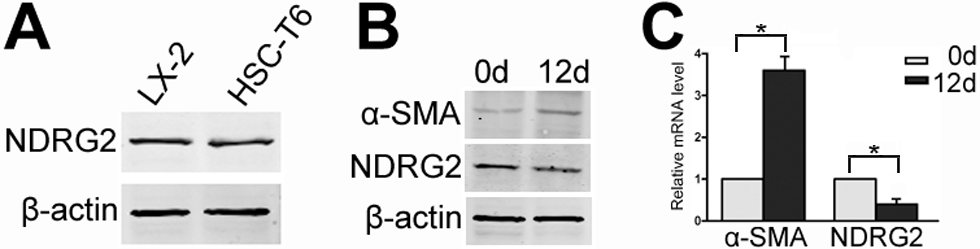

Supplement: Figure S1 — NDRG2 is decreased during HSC-T6 activation. (A) NDRG2 was expressed in LX-2 and HSC-T6 cells. Western blotting (B) and real-time PCR (C) were used to detect the expression of α-SMA and NDRG2 in HSC-T6 cells grown to confluence and then replated on 100 mm dishes for 12 days. β-actin was used as a loading control. All experiments were performed independently in triplicate. *P<0.05 between compared groups. (TIF) [file pone.0027710.s001.tif]

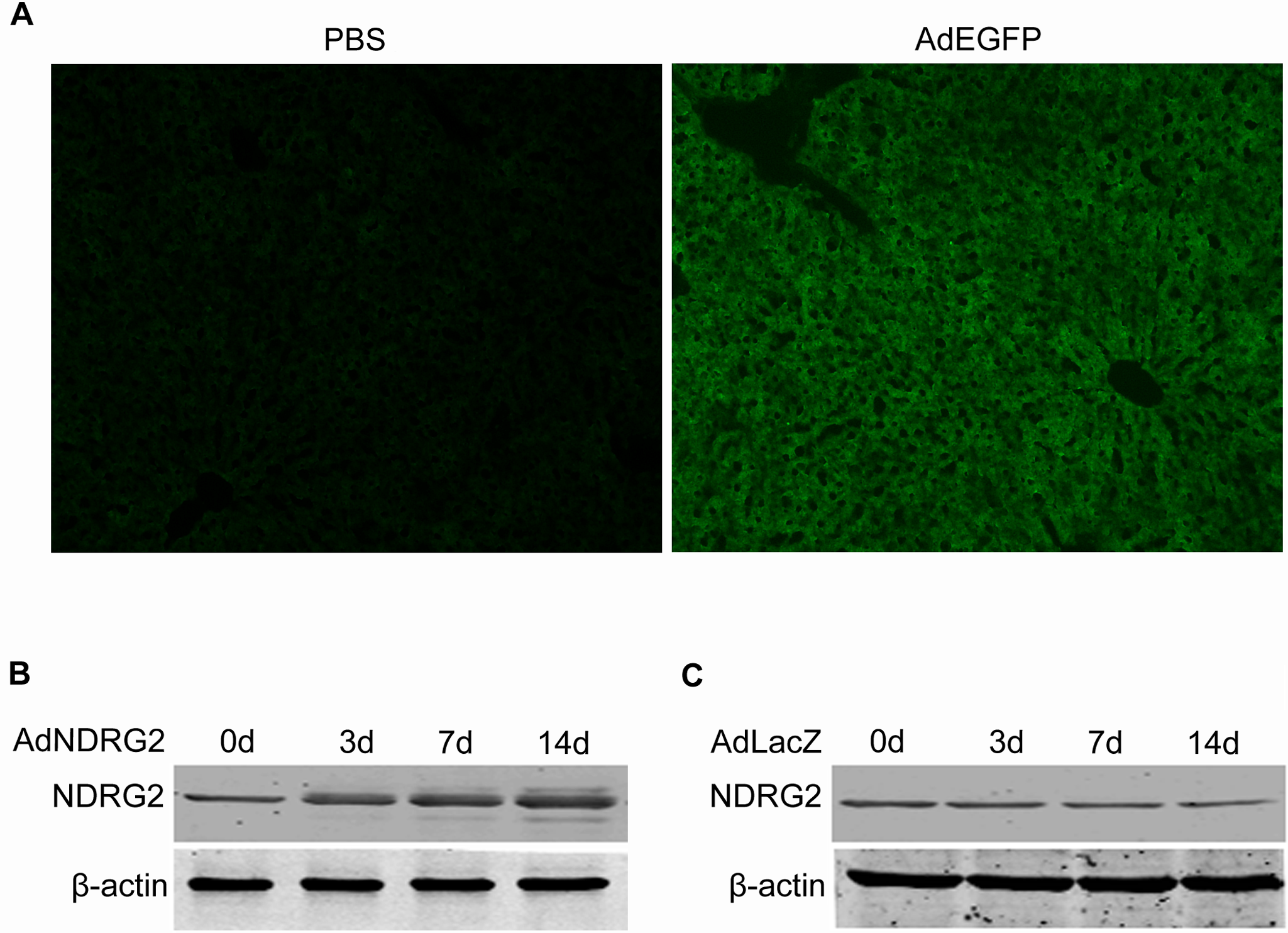

Supplement: Figure S2 — Adenovirus has a high infection efficiency and significantly enhanced NDRG2 expression in rat liver. (A) Rats were injected with PBS or a single dose (4×10^9^ PFU) of AdEGFP via the tail vein. Then, 48 hours after injection, rats were sacrificed and liver samples were fixed with neutral buffered 4% paraformaldehyde overnight at 4°C in the dark. Liver sections (10 µm thick) were prepared on a cryostat microtome and fixed to glass slides. Sections were examined directly under a fluorescent microscope (100×). (B) Rats were treated with a single dose (4×10^9^ PFU) of AdNDRG2 or AdLacZ via the tail vein. Over the next 14 days, rats were sacrificed at the time points indicated. Liver samples were collected and proteins were extracted directly. NDRG2 expression was examined by Western blotting. β-actin was used as a loading control. (TIF) [file pone.0027710.s002.tif]

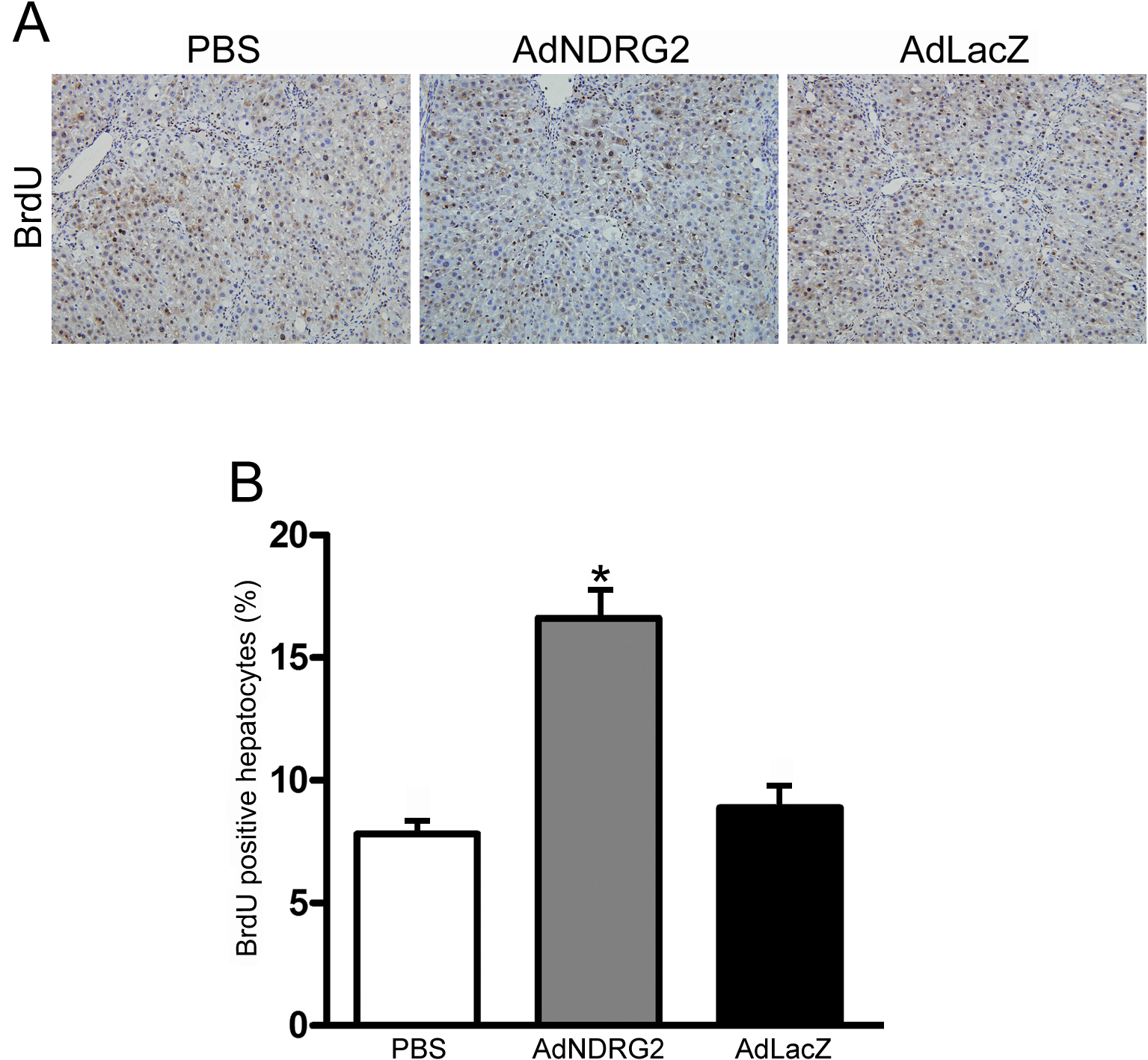

Supplement: Figure S3 — NDRG2 promotes hepatocyte proliferation. (A) Immunohistochemistry staining was used to determine the expression of BrdU (200×) in DMN-induced fibrotic rat livers following PBS or adenovirus treatment. (B) Semiquantitative analysis of BrdU staining. *P<0.05 compared with the PBS or AdLacZ group. (TIF) [file pone.0027710.s003.tif]
